# Nanostructured Lipid Carriers as Robust Systems for Lupeol Delivery in the Treatment of Experimental Visceral Leishmaniasis

**DOI:** 10.3390/ph16121646

**Published:** 2023-11-23

**Authors:** Jéssica Adriana Jesus, Thays Nicolli Fragoso da Silva, Ilza Maria Oliveira Sousa, Aurea Favero Ferreira, Márcia Dalastra Laurenti, Paulo Cardoso da Costa, Domingos de Carvalho Ferreira, Luiz Felipe Domingues Passero

**Affiliations:** 1 Institute of Biosciences, São Paulo State University (UNESP), Praça Infante Dom Henrique, s/n, São Vicente 11330-900, SP, Brazil; jessica.dolly@hotmail.com; 2 Institute for Advanced Studies of Ocean, São Paulo State University (UNESP), Rua João Francisco Bensdorp, 1178, São Vicente 11350-011, SP, Brazil; 3 Laboratório de Patologia Clínica, Departamento de Patologia, Hospital das Clinicas, Faculdade de Medicina, Universidade de São Paulo, Av. Dr. Arnaldo, 455, Cerqueira César, São Paulo 05403-000, SP, Brazil; thays.nicolli@gmail.com (T.N.F.d.S.); aurea.favero@gmail.com (A.F.F.); mdlauren@usp.br (M.D.L.); 4 Faculty of Medical Sciences, University of Campinas-UNICAMP, Rua Tessália Vieira de Camargo, 126, Campinas 13083-871, SP, Brazil; ilzamo.sousa@gmail.com; 5 UCIBIO, REQUIMTE, MEDTECH, Laboratory of Pharmaceutical Technology, Department of Drug Sciences, Faculty of Pharmacy, University of Porto, Rua Jorge de Viterbo Ferreira, 228, 4050-313 Porto, Portugal; pccosta@ff.up.pt (P.C.d.C.); domingos@ff.up.pt (D.d.C.F.); 6 Associate Laboratory i4HB—Institute for Health and Bioeconomy, Faculty of Pharmacy, University of Porto, 4050-313 Porto, Portugal

**Keywords:** nanostructured lipid carriers, drug delivery system, lipid nanocarriers, lupeol, visceral leishmaniasis

## Abstract

Leishmaniasis is a neglected tropical disease that affects millions of people around the world. Available therapy causes severe side effects, has unacceptable prices for some specific formulations, and the existence of drug-resistant parasites limits the use of the currently available arsenal of antiparasitic drugs. Therefore, natural products serve as one of the main sources to develop new and effective alternative drugs against leishmaniasis. In this sense, the present study evaluated the potential of the triterpene Lupeol (Lu) entrapped in nanostructured lipid carriers (NLCs) for the treatment of experimental visceral leishmaniasis. The therapeutic efficacy of Lu or Lu entrapped in NLC (Lu-NLC) was investigated in golden hamsters infected with *Leishmania (Leishmania) infantum*. Lu-NLC presented a mean particle size of 265.3 ± 4.6 nm, a polydispersity index of <0.25 and a zeta potential of −37.2 ± 0.84 mV; the efficacy of encapsulation was 84.04 ± 0.57%. Studies on hamsters showed that Lu-NLC (5 mg/kg) administered intraperitoneally for 10 consecutive days caused a reduction of 99.9% in the number of parasites in the spleen and liver compared to the untreated infected control. On the contrary, Lu-treated animals (5 mg/kg) had 94.4 and 90.2% less parasites in the spleen and liver, respectively, than the infected group. Additionally, a significant preservation of splenic and hepatic tissues was observed in animals treated with Lu-NLC or Lu. Furthermore, Lu-NLC-treated animals produced high levels of anti-*Leishmania* IgG2 isotype. These data indicate that NLC potentialized Lu efficacy in experimental visceral leishmaniasis. This work suggests that Lu and nanoformulations carrying this compound may be considered as an important tool to be included in the alternative therapy of leishmaniasis.

## 1. Introduction

Leishmaniasis is an infectious zoonotic disease with worldwide distribution, caused by pathogenic protozoa of the genus *Leishmania* and transmitted by sandflies, with a global prevalence of approximately 12 million cases and around 350 million people living in areas at risk of transmission [1]. It is estimated that 0.7 to 1 million new cases of leishmaniasis are reported per year in almost 100 endemic countries [2]. Human infection is mediated by about 22 species that can take two main clinical forms such as cutaneous (CL) and visceral leishmaniasis (VL) [3]. Despite the high number of CL, VL has been considered as the most life-threatening clinical form of this infectious disease, with an incidence of 400,000 cases per year and a mortality rate of up to 95% if untreated and up to 10% even when treated [4]. These data highlight the importance of an early and effective method to diagnose leishmaniasis based on classical [5] as well as new methods [6].

VL can be associated with an acute or chronic infection characterized by fever, anemia, and spleen and liver edema [7]. Treatment of leishmaniasis has been based mainly on the use of pentavalent antimonials, pentamidine, amphotericin B, and its liposomal formulation, paromomycin, and miltefosine. Despite the high activity on parasites, these medicines are toxic or costly in the case of liposomal formulations containing amphotericin B. Furthermore, parasite resistance has progressively increased, making the therapeutic regimen even more challenging [8,9,10]. In this sense, it is urgent to characterize new active compounds, such as those identified in plants.

Lupeol (Lu) is a biologically active triterpenoid produced and accumulated by some species of plants, such as *Bauhinia variegata*, *Euphorbia resinifera,* and *Sterculia villosa*, which has important pharmacological effects [11]. Furthermore, plants capable of producing Lu have been observed to exhibit leishmanicidal activity, such as *Millettia richardiana* [12], *Kleinia odora* [13], and *Platonia insignis* [14]. Studies conducted with purified Lu showed that this triterpene was capable of killing promastigote forms of *L. (L.) infantum* with high selectivity, and this effect was associated with ultrastructural and physiological changes in the mitochondria–kinetoplast complex [15]. Furthermore, Lu was active in decreasing the number of intracellular amastigote forms of *L. (L.) amazonensis* [14], *L. (L.) donovani* [14], and *L. (L.) infantum* [13], suggesting a multispectral action on *Leishmania* sp. Furthermore, this triterpene can activate macrophages, which is an interesting feature in potentializing the leishmanicidal activity of lupeol.

Despite the leishmanicidal and immunomodulatory activity triggered by Lu, very few studies demonstrated the therapeutic potential of this molecule in experimental studies. The first report showed that BALB/c mice infected with *L. (L.) donovani* exhibited a low splenic parasitism associated with a significant production of Th1 cytokines [16]; furthermore, golden hamsters infected with *L. (L.) infantum* and treated with Lu showed a dramatic decrease in the number of parasites in the spleen and liver, associated with a significant increase in the expression of the IFN-γ and iNOS genes; furthermore, these animals do not have alterations in the biochemical parameters of the blood [15].

Additionally, these studies highlighted that Lu was able to eliminate *Leishmania* sp. and activate macrophages, leading to significant efficacy in visceral leishmaniasis. Therefore, it is believed that Lu is an interesting molecule for developing new active drugs or approaches for the treatment of visceral leishmaniasis. However, it is still important to potentialize the activity of such a triterpene, and in this regard, nanoformulations appear as a promising alternative.

The development of lipid nanoparticles emerges as a promising alternative for the treatment of leishmaniasis, given that many of the substances that are shown to be active in in vivo studies often have low solubility in physiological diluents, such as the triterpene Lu, which in fact may limit its bioavailability in vivo. Thus, the development of a drug delivery system would certainly improve the therapeutic efficacy of molecules with low solubility, as is the case for ursolic acid [17] and Lu.

Lipid nanoparticles have the ability to overcome biological barriers, maximize therapeutic effect, and allow drug release at the desired specific location with reduced toxic effects compared to the free drug [18,19,20,21]. Furthermore, these nanocarriers provide a passive targeting mechanism due to the natural tendency of macrophages to phagocytose these nanoparticles without any surface modification [22]. This mechanism makes nanoparticles an attractive vector for passive targeting of poorly soluble substances with leishmanicidal action [23,24]. Therefore, NLCs as a drug delivery platform represent an opportunity to improve leishmaniasis therapy, especially using Lu triterpene whose reports have already shown that different species of *Leishmania* are susceptible to this triterpene and can therefore be considered as an alternative drug in visceral leishmaniasis therapy [16,25,26]. 

Considering that the available leishmaniasis treatment options cause severe adverse effects in humans and some of them are ineffective in controlling parasite spreading, especially in infections caused by drug-resistant strains of *Leishmania*, it would be significant to replace them with a new, effective, and nontoxic treatment. In this context, this article shows, for the first time, that Lu entrapped in nanostructured lipid carriers exhibited a potent and significant leishmanicidal activity in experimental visceral leishmaniasis compared to treatment performed only with Lu or AmB, suggesting that this formulation can be considered as an interesting alternative to conventional therapy.

## 2. Results

### 2.1. Physical–Chemical Characterization of NLC

Data on mean particle size (PS), polydispersity index (PDI), zeta potential (ZP), and encapsulation efficacy (EE) are shown in Table 1. The mean size of Lu-NLC and NLC was around 267 nm. Nanoformulation PDIs were similar (PDI < 0.2); ZP of Lu-NLC was significantly lower (−37.2 mV) than the ZP of NLC (−26.5 mV). The EE of Lu was 84.04%. 

**Figure 1:**
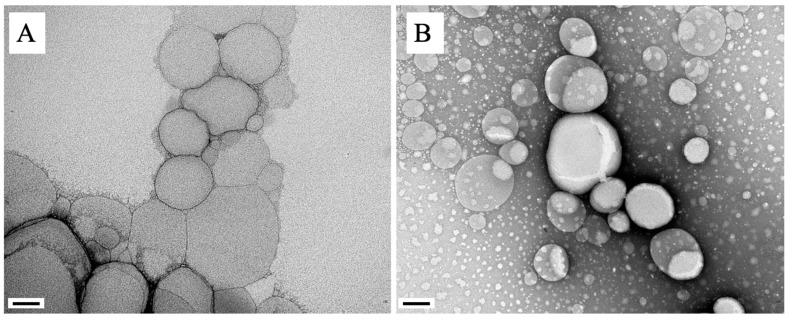
Transmission electron microscopy of the NLC (**A**) and Lu-NLC (**B**). Magnification of 100,000×. Scale: 100 nm.

Transmission electron microscopy revealed that Lu-NLC and NLC presented spherical morphology with uniform shape and smooth surface (Figure 1), corroborating the results shown in Table 1. Furthermore, it is possible to observe that when Lu is encapsulated in NLC, no significant changes in the morphology of the nanoparticles were observed (Figure 1A,B). 

### 2.2. Analysis of the Therapeutic Potential of Lu-NLC

**Figure 2:**
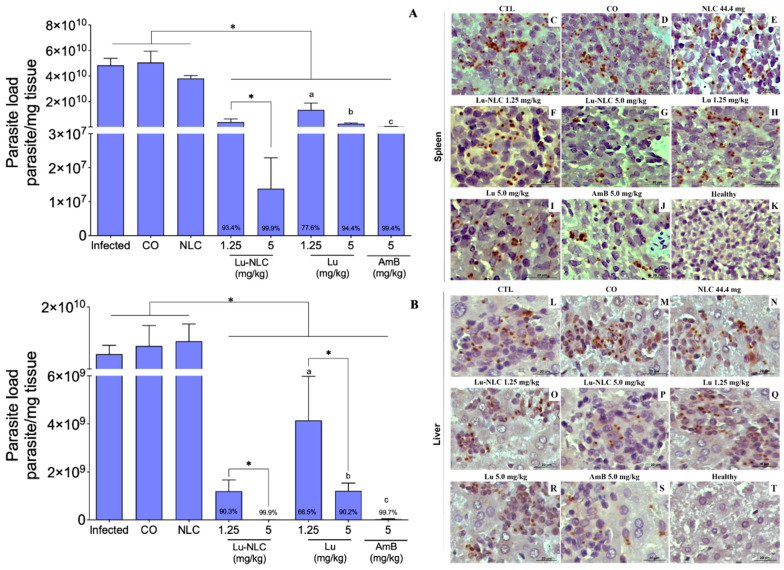
Golden hamsters with experimental visceral leishmaniasis were treated with Lu-NLC or Lu; after 10 days of treatment, tissue parasitism was determined with limiting-dilution assay. Parasite burden in the spleen (**A**) and liver (**B**) of animals treated with Lu-NLC at 1.25 and 5.0 mg/kg; Lu at the same concentrations, or with 5.0 mg/kg AmB. Histological sections stained using the immunohistochemistry technique illustrate amastigote forms (stained in dark brown) in the spleen (**C**–**J**) and liver (**L**–**S**) of the untreated control group (**C**,**L**), as well as in Lu-NLC (**F**,**G**,**O**,**P**), Lu (**H**,**I**,**Q**,**R**), animals treated with AmB (**J**,**S**) or healthy animals control group (**K**,**T**) (magnification 400; scale bar: 20 μm). * *p* < 0.05 indicates statistical significance compared to the untreated infected group. ^a,b^ *p* < 0.05 in comparison to animals treated with 1.25 or 5 mg/kg Lu-NLC. ^c^ *p* < 0.05 in comparison to animals treated with 5 mg/kg Lu-NLC.

Compared to the infected control, animals treated with Lu-NLC at 1.25 and 5 mg/kg reduced splenic parasitism by 93.4 and 99.9% (Figure 2A) and hepatic parasitism by 90.3 and 99.9%, respectively (Figure 2B). Animals treated with Lu at 1.25 and 5 mg/kg reduced splenic parasitism by 77.6 and 94.4% and hepatic by 66.5 and 90.2%, respectively. Furthermore, it was observed that animals treated with Lu-NLC, exhibited significantly lower splenic and hepatic parasitism compared to Lu (Figure 2A,B). Animals treated with amphotericin B (AmB) also showed a significant reduction in splenic and hepatic parasites; however, animals treated with 5.0 mg/kg of Lu-NLC showed a lower parasitic load compared to animals treated with AmB (Figure 2A,B). Furthermore, AmB was more active in eliminating tissue parasites than Lu. Animals injected with NLC or corn oil (CO)—Lu diluent—did not exhibit a significant reduction in the number of tissue parasites in comparison with the infected control.

Histological sections of the spleen and liver showed a high number of amastigotes in the infected control (Figure 2C,L), CO (Figure 2D,M) or NLC-treated control groups (Figure 2E,N), but a low number of parasites in animals treated with 1.25 and 5.0 mg/kg of Lu and lower in animals treated with Lu-NLC (Figure 2F–I,O–R) as well as AmB (Figure 2J,S).

### 2.3. Histopathological Alterations in the Spleen and Liver of Animals Treated with Lu-NLC or Lu

**Figure 3:**
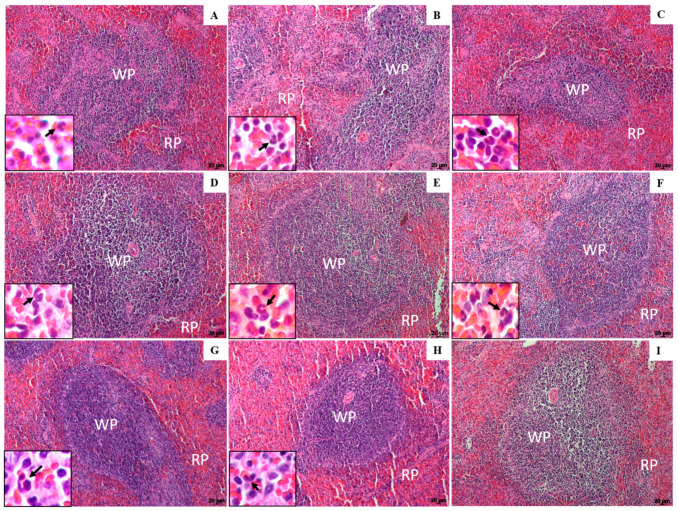
Histological sections of the white pulp (WP) and red pulp (RP) of the spleen of golden hamsters. Spleen histological sections of infected controls ((**A**)—infected control, non-treated; (**B**)—infected and treated with corn oil; (**C**)—infected and treated with NLC); animals treated with 1.25 and 5.0 mg/kg Lu loaded in NLC ((**D**,**E**), respectively), animals treated with 1.25 and 5.0 mg/kg Lu ((**F**,**G**), respectively) or AmB (**H**). The histological section of the spleen from non-infected animals is shown in image (**I**). The insets show polymorphonuclear cells (arrows) of the histological sections of the spleen. Magnification of 40×; scale bars: 20 μm (**A**–**I**).

Histological sections of the spleen from the infected control, CO- and NLC-injected groups showed hyperplasia of frequently parasitized macrophages in the red pulp (RP) area. These macrophages were arranged in granulomas. Furthermore, a high frequency of polymorphonuclear cells was observed, demonstrating a greater severity of the disease in the control groups (Figure 3A–C, black arrow).

In animals treated with different doses of Lu-NLC (1.25 and 5.0 mg/kg), a greater preservation of white pulp (WP) was observed, suggesting a better host immune response after treatment (Figure 3D,E). The expansion of RP with polymorphonuclear cells (inset) and the presence of macrophage nodules were also observed, but in smaller numbers, which in turn exhibited a reduced number of intracellular parasites. Animals treated with Lu (1.25 and 5.0 mg/kg) also exhibited preservation of WP compared to the control; however, macrophage nodules in RP (Figure 3F,G) were observed more frequently than in animals treated with Lu-NLC. Compared to the infected control group (Figure 3A), hamsters treated with 5.0 mg/kg of AmB (Figure 3H) showed preservation of WP; however, an expansion of the RP marked by the presence of macrophage nodules and polymorphonuclear cells was observed (inset in Figure 3H). Histological sections of the spleens of healthy animals showed a normal histological appearance, well-preserved white and red pulps, as shown in Figure 3I.

**Figure 4:**
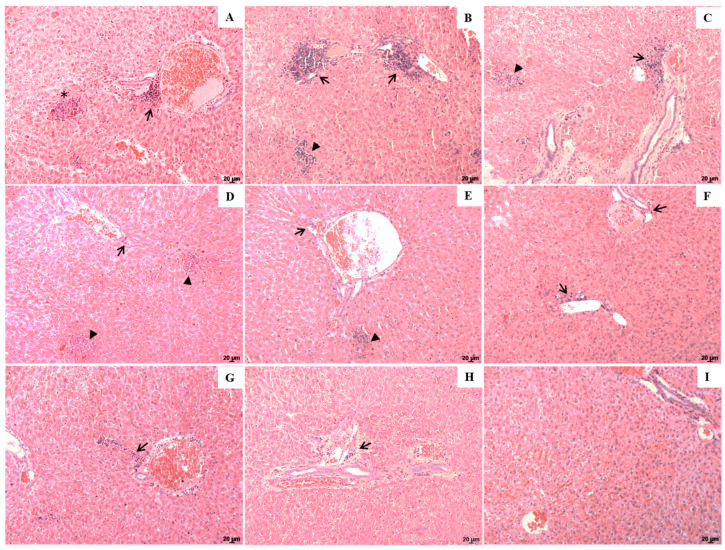
Photomicrographs of histological sections of the liver of golden hamsters. Liver histological sections of infected controls ((**A**)—infected control, non-treated); (**B**)—infected animals injected with corn oil; (**C**)—infected and treated with NLC); animals treated with 1.25 and 5.0 mg/kg Lu-NLC ((**D**,**E**), respectively), animals treated with 1.25 and 5.0 mg/kg Lu ((**F**,**G**), respectively); (**H**)—animals treated with 5.0 mg/kg AmB. The histological section of the liver from healthy animals is shown in image (**I**). * indicates a focus of hepatocellular necrosis; black arrow indicates the region with inflammatory infiltrate, and the arrowhead indicates nodules in the parenchyma of the histological sections of the liver. Magnification of 40×; scale bars: 20 μm (**A**–**I**).

In histological sections of the liver, the main changes detected in all groups were the presence of a periportal inflammatory infiltrate, characterized by the accumulation of mononuclear cells, mainly macrophages, lymphocytes and plasma cells. Granulomas in the portal space and in parenchyma were also observed (Figure 4A–H). Furthermore, all infected groups presented hyperplasia and hypertrophy of Kupffer cells, sometimes parasitized, as shown in immunohistochemistry reactions (Figure 2); however, parasitism and nodule formation were lower in Lu-NLC treated groups (Figure 4D,E), than in Lu (Figure 4F,G) and AmB-treated groups (Figure 4H). Hamsters treated with 5.0 mg/kg of AmB also showed preservation of the periportal spaces, although the inflammatory areas were verified but to a lesser extent compared to the infected control group (Figure 4H), reduced number of nodules was also evidenced in the parenchyma. Possibly, all these differences are related to the number of parasites, considering that a high number of parasites will cause a significant influx of inflammatory cells that is associated with tissue destruction; in contrast, a low number of parasites will induce a low to moderate influx of inflammatory cells, and in turn, tissue will be more preserved compared to a highly infected organ. Healthy animals presented preserved periportal spaces with normal hepatocytes without any degeneration aspect (Figure 4I).

### 2.4. Analysis of Antibody Production

**Figure 5:**
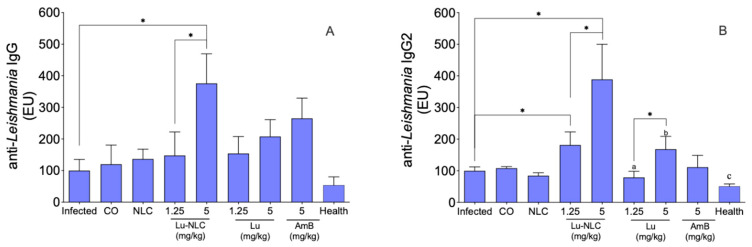
Anti-*Leishmania* IgG (**A**) and IgG2 (**B**) levels were analyzed in the serum of golden hamsters infected with *L. (L.) infantum*, treated or not with 1.25 or 5.0 mg/kg Lu-NLC or Lu. As a standard treatment, hamsters were treated with AmB intraperitoneally (5.0 mg/kg). * *p* < 0.05 indicates statistical significance. ^a,b^ *p* < 0.05 animals treated with Lu VS animals treated with Lu-NLC in the same dose. ^c^ *p* < 0.05 health animals VS treated with Lu-NLC.

The humoral immune response demonstrated that only animals treated with 5.0 mg/kg Lu-NLC produced significant amounts of anti-*Leishmania* IgG compared to the untreated infected control group and animals treated with 1.25 mg/kg of Lu-NLC (Figure 5A). Increased IgG levels were highly associated with elevated IgG2 levels in groups treated with 1.25 and 5 mg/kg Lu-NLC (Figure 5B) compared to all control groups and the respective groups of animals treated with Lu (*p* < 0.05). Furthermore, animals treated with 5 mg/kg of Lu-NLC were found to produce higher levels of IgG2 than animals treated with 1.25 mg/kg Lu-NLC (*p* < 0.05). Animals treated with 5.0 mg/kg Lu-NLC showed high levels of IgG2 compared to animals treated with Lu at the same dose (Figure 5B). Animals treated with AmB did not change IgG and IgG2 levels compared to the infected control groups.

## 3. Discussion

Targeted drug delivery is a key application of nanotechnology in medicine that has aroused great interest in the treatment of neglected parasitic diseases, given the advantages that different transport systems can confer during treatment, including the reduction in side effects while increasing the effectiveness and selectivity of drugs [19,24]. In this sense, nanostructured lipid carriers associated with Lu were prepared to increase the efficiency of this promising antiparasitic molecule in the treatment of visceral leishmaniasis. 

The mean particle size and PDI values found for NLCs prepared using hot high pressure homogenization revealed a monodisperse distribution of nanoparticles of small mean size (<267 nm) and with physical–chemical aspects indicative of stable systems, with very negative ZP values, homogeneously dispersed particles with low PDI (lower than 0.25), which can be explained by the stability of wax (cetyl palmitate) and the low molecular weight of the surfactant (tween 80) used in the preparation of nanoformulations [27], suggest that this nanocarrier is capable of circulation through tissues [28]. These results corroborated data found in other studies conducted with NLC [29,30], demonstrating that such nanoparticles have a similar range of negative zeta values with physical stability over 1 year of storage, that in fact justify the addition of suitable steric stabilizers. Additionally, the formulation showed a high encapsulation capacity of Lu (84.04 ± 0.57%), indicating that the high lipophilicity of Lu favored the interaction of the triterpene with the constituent lipids of the particles. Furthermore, transmission electron microscopy validated the nanometer size range and confirmed that the particles displayed globular shape, clear margins with no signs of aggregation, suggesting that the produced nanoformulations were stable. The analysis of the polymorphic form of formulation is essential, as the nature of these components (crystalline or amorphous) influences the properties of the formulation [31,32]. Taking into account the successful incorporation of Lu in NLC, its therapeutic potential was analyzed in experimental VL. 

In experimental animals, both Lu-NLC and Lu were effective in treating golden hamsters with experimental visceral leishmaniasis, since a dramatic and significant reduction in splenic and liver parasitisms was observed compared to the control; moreover, Lu-NLC at 5 mg/kg was even more active in eliminating parasites in the spleen and liver than AmB. In fact, it should be considered as an important achievement, considering that AmB is toxic to humans and experimental animals [33].

In the specific case of this experiment, it was observed that Lu-treated animals (1.25 and 5 mg/kg) received a cumulative dose of 2.0 and 8.0 mg of Lu, respectively, and presented reductions in parasitism of 77.6 and 94.4% in the spleen and 66.5 and 90.2% in the liver, respectively. On the other hand, animals treated with 5.0 mg/kg of AmB received a total cumulative dose of 8.0 mg, and this amount of AmB reduced the splenic and hepatic parasitisms by 99.4 and 99.7%, respectively. Though considered as an effective treatment due to its high therapeutic potential, this dose of AmB can induce serious side effects in the host, as previously demonstrated [33]. Furthermore, the results presented here corroborate the findings shown by Das [25] and Kaur [16], who showed that Lu reduced the number of parasites in the spleen and liver of experimental animals with visceral leishmaniasis without causing toxic events in the host.

Furthermore, it was observed that Lu treatment became more effective when encapsulated in NLC, which even at lower doses eliminated a large number of parasites in the spleen and liver; this is probably due to the fact that when the drug is encapsulated in NLC an effective increase in its bioavailability should occur, improving its effectiveness in the treatment of visceral leishmaniasis. In addition, it is important to highlight the superiority of the treatment carried out with Lu-NLC over that with Lu. Comparatively, it was observed that treatment with Lu at a dose of 1.25 mg/kg (total cumulative dose of 2.0 mg Lu) reduced parasitism by 77.6% in the spleen and 66.5% in the liver, while the same dose of Lu administered as a nanocarrier reduced the parasitism in the spleen by 93.4% and 90.3% in the liver. Animals treated with 5.0 mg/kg of Lu (total dose of 8.0 mg) had a reduction of 94.4% in the parasitism of the spleen and 90.2% in the parasitism of the liver, although a potentiation in efficacy was observed when animals were treated with Lu-NLC at the same dosages; in this case, it was observed that treatment with 5.0 mg/kg of Lu-NLC reduced 99.9% of the parasitism in the spleen and liver. Therefore, such data show that encapsulation is an important strategy to maximize the biological potential of this triterpene, which is consistent with other studies demonstrating the increased efficacy of different compounds in the treatment of leishmaniasis when carried out in NLC [20,25,34,35]. Furthermore, the potentialization of Lu when entrapped in NLC can be explained considering some intrinsic characteristics of lipid nanoparticles, such as increased bioavailability of incorporated drugs and controlled drug release, furthermore, macrophages are able to phagocytosis such nanoparticles, which is an advantage because parasites preferentially infect such cells [24,36].

The potential of NLC carrying the triterpene ursolic acid [17] and the monoterpene carvacrol [37] was also demonstrated in the treatment of leishmaniasis. Such studies are in line with what we observe herein and reinforce that NLCs are promising delivery systems for the treatment of leishmaniasis and suggest that this nanosystem can improve the effectiveness of drugs when compared to those administered in free form, given that nanoparticles work as good drug carriers.

In addition to a reduced number of tissue parasites, Lu-NLC-treated animals exhibited greater preservation of the spleen and liver compared to infected controls and Lu-treated animals. In histological sections of the spleen of these animals, discrete macrophage hyperplasia was observed in the RP associated with a few macrophage nodules, suggesting that Lu-NLC treatment was highly active in preserving the splenic structure compared to infected controls, which exhibited characteristics associated with progressive and active VL [38]. 

In the liver, an inflammatory infiltrate was observed in the portal space whose intensity varied according to treatment. In this case, less intense inflammation was observed in animals treated with Lu-NLC compared to animals that received only Lu. In animals treated with Lu-NLC or AmB, the development of an inflammatory process characterized by the presence of a discrete mononuclear cell infiltrate was observed in portal spaces with low parasitism, which differed from the infected control groups (that were constituted by infected animals; animals injected with NLC or corn oil), which also presented areas of hepatocellular necrosis. Experimental studies carried out with golden hamsters infected with *Leishmania donovani* demonstrated that infection induces an intense inflammatory infiltrate the periportal space that was associated with tissue destruction and death of hepatocytes [39]. On the other hand, a low-intensity inflammatory infiltrate associated with a significant preservation of the hepatic tissue observed in Lu-NLC-treated animals suggests that this nanoformulation is highly active in treating active disease and also reinforces that Lu is an alternative to the treatment of VL. Despite the high efficacy of Lu-NLC in VL, the main limitation of its use is associated with the route of administration that should be standardized in the near future to the oral route, that is associated with a more tolerable route of drug administration. Furthermore, although available studies pointed out low or absent side effects in animal models, pharmacokinetic and pharmacodynamic studies should be performed to investigate Lu pharmacology in primates.

Although the hamster model of visceral leishmaniasis mimics a natural infection, this model has some major drawbacks, such as the difficulty in finding specific reagents to quantify bioactive interleukins. Therefore, to overcome this problem, we opt to analyze antibody levels, since there is a correlation between the IgG2 isotype and the development of a Th1 immune response and resistance in infections caused by *Leishmania* sp. [37,40,41,42]. In the present study, animals treated with Lu-NLC were found to exhibit a significant increase in anti-*Leishmania* IgG, which was highly associated with the production of the IgG2 isotype. Taking into account the development of the IgG2 and Th1 immune response axis, it is possible that Lu-NLC treatment stimulated IFN-γ production, as previously observed in BALB/c mice infected with *L. (L.) donovani* and treated with Lu [16,25]. Therefore, the humoral immune response developed by animals treated with Lu-NLC suggests that there is a tendency to induce a Th1 response in golden hamsters treated with Lu, mainly in groups treated with Lu-NLC.

Current studies on this delivery system (NLC) have provided information on possible indications and evidence of its enhanced ability to specifically deliver the drug to pathogens, penetrate host barriers, and reduce toxicity with lower dose regimens [43]. In this sense, the data obtained reinforce the potential and safety of Lu entrapped in NLC and effectively demonstrate the in vivo leishmanicidal action of Lu in this nanosystem, indicating that this triterpene can be considered an interesting molecule in the search for potential leishmanicidal agents and, when formulated in NLC, has its therapeutic activities potentialized through the targeted distribution of the drug to target organs of parasites, being a potential therapeutic alternative in the treatment of visceral leishmaniasis.

## 4. Materials and Methods

### 4.1. Materials 

For the nanoparticle production, the solid lipid cetyl palmitate was provided by Gattefossé SAS (St Priest, France), the liquid lipid miglyol 812, and polysorbate 80 (Tween^®^ 80) were purchased from Acofarma^®^ (Madrid, Spain). Lu (purity ≥ 98%) was obtained from Cayman chemical company (Ann Arbor, MI, USA) and AmB (purity ≥ 99%) from Cristalia Laboratory (São Paulo, Brazil). Acetonitrile was obtained from VWR (Radnor, PA, USA), ultrapure water (type 1, Milli-Q^®^) from EMD Millipore (Billerica, MA, USA). Cell culture media were acquired from Sigma-Aldrich (Darmstadt, Germany).

### 4.2. Preparation of NLC

The NLC and Lu-NLC were prepared using a high-pressure homogenization technique. To produce 10 g of nanoformulations, firstly, the oily phase was prepared via melting cetyl palmitate (0.7 g) and mygliol-812 (0.3 g) in a water bath at 70 °C. Subsequently, Lu (0.1 g) was added as the oily phase. The aqueous phase, composed of polysorbate 80 (0.2 g) and distilled water (q.s. 8.7 g), was then heated up to the same temperature as the oil phase and mixed with the oily component.

The emulsion was subjected to a homogenization process (Ultra-Turrax T25, with the dispersing element S 25 N—18 G IKA^®^-Labortechnik, Staufen, Germany) at a stirring speed of 10,400 rpm for 5 min. For the NLC formation, the prepared emulsion was quickly transferred to a high-pressure hot homogenizing equipment (High-Pressure Homogenizer SPCH-10, Stansted Fluid Power, Harlow, UK), and homogenized in five cycles at 600 bars, and then cooled to 25 °C in an ice bath. The samples were stored in glass bottles at 4 °C. Empty NLCs were prepared in a similar way, without adding Lu.

In all the experiments, Lu was quantified using the UltiMate 3000 HPLC apparatus (Dionex Corporation, Sunnyvale, CA, USA), with a controller, isocratic system, UV-VIS detector, and automatic injector. A reverse phase C18 column (BDS-Hypesil-C18^®^, Thermo Scientific, Waltham, MA, USA) was used with an acetonitrile mixture: water (99.9:0.01, v:v) mixture as the mobile phase, flow rate = 1.2 mL·min^−1^; Lu absorbance was detected at 210 nm [44,45].

### 4.3. Physical–Chemical Characterization of Nanoparticles

#### 4.3.1. Determination of Encapsulation Efficiency

Lu-NLC was diluted in milli-Q water (1:5, v:v), filtered in a 5 µm nitrocellulose membrane filter (Millipore, Dublin, Ireland) and diluted 1:10 (v:v) in ethanol to extract Lu from the NLC. The mixture was centrifuged at 4620× *g* at 25 °C for 15 min; the supernatant was collected and filtered through a 0.45 µm PTFE syringe filter (Millipore, Ireland) [46]. The supernatant was diluted (1:6, v:v) in an eluent solution and applied to the HPLC column. The amount of Lu released was quantified using a standard curve constructed with a known concentration of Lu (20 a 70 μg/mL). Encapsulation efficiency (EE) was determined by calculating the amount of Lu in the supernatant of the filtered formulations as follows:

(1) $$EE\%=\frac{\mathrm{Amount}\;\mathrm{of}\;\mathrm{Lu}\;\mathrm{in}\;\mathrm{the}\;\mathrm{filtered}\;\mathrm{formulation}}{\mathrm{Total}\;\mathrm{amount}\;\mathrm{of}\;\mathrm{Lu}}\times 100$$

#### 4.3.2. Determination of Size, Polydispersity, and Zeta Potential

The mean particle size (PS) and polydispersity index (PDI) were analyzed with dynamic light scattering (DLS, ZetaPALS, Brookhaven Instruments, Holtsville, NY, USA). The zeta potential (ZP) of the NLC dispersions was measured using electrophoretic light scattering (ELS) on a zeta potential analyzer (ZetaPALS, Brookhaven Instruments, Holtsville, NY, USA). Samples were diluted (1:200) in milli-Q water, resulting in a suitable scattering intensity. The samples were analyzed at room temperature, with a fixed light incidence angle of 90°; the mean hydrodynamic diameter (Z-average), PDI, and ZP were obtained by calculating the mean value of six measurements, performed in three different samples [17].

#### 4.3.3. Transmission Electron Microscopy of Lu-NLC

Transmission electron microscopy (JEOL JEM 1400, Tokyo, Japan) was used to analyze the morphology of the Lu-NLC and NLC. An aliquot of nanoparticles (10 μL) was placed on nickel gratings with Formvar mesh/carbon film (Electron Microscopy Sciences, Hatfield, PA, USA). The samples were contrasted with a 1% uranyl acetate solution. The samples were analyzed under a microscope at a voltage of 120 kV [17]. Images were recorded using a CCD digital camera Orious 1100 W Tokyo, Japan.

### 4.4. Animals 

Golden hamsters (*Mesocricetus auratus*), 8 weeks old, were obtained from Anilab (Paulinia, São Paulo, Brazil). Hamsters were housed at the Animal Facility of the Instituto de Medicina Tropical da Universidade de São Paulo (IMT-USP) and allowed to access food and water ad libitum throughout the study in a 12 h light cycle. Animals were euthanized with a lethal dose of sodium thiopental via intraperitoneal route. 

Animal studies were carried out strictly in accordance with the recommendations of the guide for the Care and Use of Laboratory Animals of the Brazilian National Council of Animal Experimentation. The protocol was approved by the Ethics Committee of Animal Experiments of the Institutional Committee for Animal Care and Use at the Medical School of São Paulo University (056/16).

### 4.5. Analysis of the Efficacy of Lu-NLC in Experimental Visceral Leishmaniasis

Golden hamsters (8 weeks old) were infected with 2 × 10^7^ *L. (L.) infantum* (MHOM/BR/72/46) promastigotes forms in stationary phase of growth via the intraperitoneal route. Healthy control group was injected with 100 μL of PBS. Infected hamsters were distributed into 9 groups, with 5 animals each. After 60 days of infection, treatment was initiated with Lu, Lu-NLC, AmB, or empty NLC. Treatment was performed via the intraperitoneal route, once a day, for 10 consecutive days. In this case, the experimental groups were organized as follows: Groups 1 and 2 were treated with Lu-NLC, containing 1.25 and 5.0 mg/kg of Lu, respectively; Groups 3 and 4 were treated with 1.25 and 5.0 mg/kg of Lu, respectively; Group 5 was treated with NLC (the equivalent amount of group 2~44.4 mg); Group 6 was treated with corn oil (CO), the diluent of Lu; Group 7 was treated with 5.0 mg/kg of AmB [47]; Group 8 was injected with PBS (Infected control group) and Group 9 was constituted of a non-infected control group, that received only PBS. NLC or Lu-NLC were solubilized in sterile PBS; Lu were solubilized in corn oil, as indicated in some studies [48,49,50]. One week after the last injection, the animals were euthanized; the sera were collected to quantify *Leishmania*-specific IgG and IgG2 via enzyme-linked immunosorbent assay (ELISA) (Southern Biotech, Birmingham, AL, USA). Fragments of the spleen and liver were collected to determine splenic and hepatic parasitism, as well as histological changes. The optimization of Lu dose was performed based on previously published articles [15,25].

#### 4.5.1. Evaluation of Parasite Burden

The splenic and hepatic parasitisms were quantified with limiting dilution assay [33]. In brief, spleen and liver fragments were collected and homogenized in S10 medium. The organ suspensions were subjected to 12 serial dilutions with four replicate wells. The number of viable parasites was determined based on the highest dilution at which promastigotes could grow after 10 days of incubation at 25 °C. Additionally, parasitism in these organs was evidenced with the immunohistochemical technique [51]. 

#### 4.5.2. Analysis of Antibody Production

Humoral immune response was analyzed using ELISA. Ninety-six-well high-binding ELISA plate (Costar, Washington, DC, USA) was sensitized with the soluble antigen of the promastigote forms of *L. (L.) infantum* (1.0 μg of protein/well) in the carbonate–bicarbonate buffer, pH 9.6 (100 mM NaHCO_3_; 6 mM Na_2_CO_3_), for 18 h at 4 °C. After this period, the plate was washed three times with PBS plus 0.05% Tween 20 (PBST)and nonspecific binding blocked with 10% skimmed milk diluted in PBS for 120 min at 37 °C. The plate was washed three times with PBST, and 100 μL of the animal sera (1:1000) were added, and the reaction was incubated for 60 min at 37 °C. Three washes with PBST were performed, and mouse anti-hamster IgG (1:1000) or anti-hamster IgG2 (1:16,000) conjugated with alkaline phosphatase or horseradish peroxidase (Southernbiotech, Washington, DC, USA) at a dilution of 1:10,000 and 1:16,000, respectively, were added to the wells for 60 min at 37 °C. The plate was washed with PBST, and the substrates p-nitrophenyl phosphate—p-NPP—(for IgG secondary antibody) or 3.3′, 5.5′ tetramethylbenzidine—TMB—(for IgG2 secondary antibody) (B&D, Roseburg, OR, USA) were added to the wells during 15 min. After this period, the reaction using TMB was blocked by adding 50 μL/well of sulfuric acid (H_2_SO_4_), 2N. The plate was read in an ELISA reader at 450 in ELISA reactions employing p-NPP and at 405 nm for reactions using TMB. Sera from animals chronically infected with *L. (L.) infantum* and healthy animals were used as positive and negative control of the reactions, respectively.

### 4.6. Statistical Analysis

All experiments were repeated at least three times, and the values obtained were expressed as mean ± standard error. Statistical analyses were performed using GraphPad Prism 9.0 software, and the ANOVA statistic tests were used to analyze the differences between groups. Statistical significance was established at *p* < 0.05.

## 5. Conclusions

In conclusion, the results shown in this article demonstrated that nanostructured lipid carriers containing Lu were produced with acceptable physical features and high efficacy of encapsulation. Furthermore, it was observed that Lu entrapped in NLC exhibited superior efficacy in eliminating amastigote forms in the spleen and liver of hamsters with experimental VL compared to the treatment performed with Lu. Furthermore, Lu-NLC-treated animals showed only minimal histopathological changes in the spleen and liver compared to controls, which were strongly associated with an increase in IgG2 isotype levels. Therefore, these data show, for the first time, that Lu-NLC is highly effective in the treatment of VL and should be considered as a significant formulation to be used in the treatment of VL.

## Figures and Tables

**Table 1:** Mean particle size (PS), polydispersity (PDI), zeta potential (ZP) and efficacy of encapsulation (EE) of nanostructured lipid carriers (NLCs) or Lu-containing NLC.

Nanoparticle	PS (nm)	PDI	ZP (mV)	EE (%)
NLC	266.3 ± 2.6	0.16 ± 0.013	−26.5 ± 1.18	
Lu-NLC	265.3 ± 4.6	0.21 ± 0.011	−37.2 ± 0.84	84.04 ± 0.57

## Data Availability

Data is contained within the article.
